# How do anxiety and stress impact the performance of Chinese doctoral students through self-regulated learning?—A multi-group analysis

**DOI:** 10.3389/fpsyg.2023.985379

**Published:** 2023-02-03

**Authors:** Zhen Li, Jinyan Huang, Shahbaz Hussain, Tiantian Shu

**Affiliations:** ^1^Zhejiang Fashion Institute of Technology, Ningbo, China; ^2^School of Teacher Education, Jiangsu University, Zhenjiang, China

**Keywords:** Chinese doctoral students, anxiety, stress, self-regulated learning, structural equation modeling

## Abstract

**Introduction:**

Highly accomplished doctoral students may suffer when they cannot manage their performance due to the crippling effects of anxiety and stress. This is even more likely to occur in the highly charged setting of competitive research. Using a structural equation modeling approach, this study examined how anxiety and stress impact the performance of Chinese doctoral students through self-regulated learning.

**Methods:**

A total of 491 doctoral students and recent completers representing 112 universities in China participated in this study. A 42-item five-point Likert scale survey was used to measure participants’ perceived anxiety (emotional and physical reactions), stress (study- and research-related stress), self-regulated learning, and performance (task and contextual performance) in their doctoral studies. Specifically, the extent to which participants’ self-regulated learning mediated the influence of anxiety and stress on their task performance and contextual performance in their doctoral studies, as well as significant structural equation modeling differences across demographic variables of gender (i.e., male versus female), major (i.e., arts versus sciences), status (i.e., individuals pursuing a doctoral degree versus recent completers), and age (i.e., 30 and younger versus over 30) were examined.

**Results and Discussion:**

The results indicated that self-regulated learning considerably affected task and contextual performance; stress had a considerable direct effect on task and contextual performance; the indirect influence of stress on task and contextual performance via self-regulated learning was significant; and there was a significant structural equation modeling difference between arts and sciences doctoral students. Educational implications are discussed.

## 1. Introduction

Doctor education is an essential step in one’s academic career. It is often considered the most challenging and rewarding step (Appel and Dahlgren, 2003; Jomaa and Bidin, 2017; Hemmati and Mahdie, 2020; Darley, 2021). Related research has shown that doctoral students experience both anxiety and stress in their doctoral studies, which would adversely affects their learning and performance (Bolliger and Halupa, 2012; Mattocks and Briscoe-Palmer, 2016; Liu and Abliz, 2019; Liu et al., 2019). Their anxiety is manifested as emotional and physical reactions (Zhang, 2016; Liu and Abliz, 2019; Liu et al., 2019). Emotional reactions include sadness, loneliness, lack of motivation, focus, and confidence; and physical reactions have fatigue, difficulty sleeping, and health problems (Janta et al., 2014; Barry et al., 2018; Liu and Abliz, 2019). Moreover, their stress comes from study and research (Barry et al., 2018; Liu and Abliz, 2019; Liu et al., 2019). For example, doctoral students feel pressure to maintain high levels of academic achievement and publish research papers (Liu and Abliz, 2019; Jones-White et al., 2020).

Although most doctoral students experience anxiety and stress in their doctoral studies, they have developed corresponding learning strategies and become committed learners who self-regulate their learning (Bolliger and Halupa, 2012; Zhang, 2016; Levecque et al., 2017; Jones-White et al., 2020). Research has indicated that students’ self-regulated learning significantly influences their learning outcomes (Janta et al., 2014; Zhang, 2016; Liu and Abliz, 2019).

During the past couple of decades, there has been an increase in the number of doctoral programs and students studying for doctoral degrees in Chinese higher education (Liu and Abliz, 2019; Liu et al., 2019; Wang and Byram, 2019). Research on Chinese doctoral students has also examined the effects of anxiety and stress on their doctoral learning performance and outcomes (Liu et al., 2019; Liu and Abliz, 2019) as well as their self-regulated learning in their doctoral studies (Zhang, 2016; Liu and Abliz, 2019; Liu et al., 2019). However, existing literature has little debate about the sorts and significance of self-regulated learning in the anxiety, stress, and performance relationship, which still needs investigation in the Chinese higher education context. So, the present study tried to explore the following research questions:

- What is the impact of anxiety and stress on Chinese doctoral students’ task and contextual performance?
- How self-regulated learning mediates the relationship between anxiety and stress and Chinese doctoral students’ task and contextual performance?
- Are there significant structural equation modeling differences across demographic variables of gender (i.e., male versus female), major (i.e., arts versus sciences), status (i.e., individuals pursuing a doctoral degree versus recent completers), and age (i.e., 30 and younger versus over 30)?

This study investigates how anxiety and stress affect task and contextual performance through self-regulated learning. It differs in several respects from earlier studies. Prior research mainly concentrated on the impact of anxiety and stress as unidimensional constructs on performance. In contrast, this work has reconciled the disparate findings by utilizing distinctive dimensions of anxiety (emotional and physical reactions) and stress (study and research-related stress) on task and contextual performance through self-regulated learning in Chinese doctoral students. The foundation of self-regulated learning in the higher education spectrum is laid by the current research’s first significant contribution, demonstrating its applicability and importance in this context. In particular, self-regulated learning is effectively explained in terms of how doctoral students react to anxiety and stress and how it influences their performance, which aids in fortifying the suggested model. Secondly, it indicates a mediation model to examine the critical relationships between self-regulated learning, anxiety, stress, and performance. This study makes a methodological addition using the partial least squares technique to provide reliable estimates for the suggested model. Thus, the study’s findings can offer some helpful recommendations for academia and policymakers to enhance Chinese doctoral students’ learning performance through self-regulated learning strategies in higher education.

## 2. Literature review and hypotheses development

### 2.1. The processing efficiency theory: An underpinning theory

The process efficiency theory (PET) was first proposed and then extended by Calvo and Eysenck (1992), and the purpose of PET was to explain how state anxiety affects performance. However, it was meant to be more relevant to the performance of tasks, to high anxiety in general populations rather than clinical groups, and to test or evaluate stress circumstances. According to the processing efficiency theory, trait anxiety and situational threat or stress interact to determine state anxiety. Additionally, it is considered that individual differences in internal processing and performance are often determined by the degree of state anxiety. The core tenet of the PET is that anxiety has an impact on performance and processing and that this impact is moderated by control or self-regulatory mechanism (Calvo and Eysenck, 1992). So, the theoretical framework of the present study followed the PET, which showed how doctoral students’ state anxiety and stress affect their performance and how this whole process is mediated by self-regulation learning, another innovation of the study. Previous studies have been conducted on anxiety, stress, and performance (Ganley et al., 2021; Hong et al., 2021; Johnson et al., 2021; Mendoza et al., 2021; Micheal et al., 2021; Vincent et al., 2021; Wolters and Brady, 2021; Caviola et al., 2022) on college students primarily in mathematics, but these constructs are unexplored in doctoral programs settings in China.

### 2.2. Students’ challenges in the doctoral program around the globe

Doctoral education is an integral part of higher education. Many researchers have investigated the learning challenges faced by doctoral students from different countries (e.g., England, America, China, Finland, and Australia) during the past couple of decades (Jiao et al., 2008; Han, 2012; Pyhältö et al., 2012; Sakurai et al., 2012; Gardner, 2013; Vekkaila et al., 2013; Bronkhorst and de Kleijn, 2016; Hu et al., 2016; Löfström and Pyhältö, 2017; Zhu et al., 2017; Laufer and Gorup, 2019; Liu and Abliz, 2019; Hemmati and Mahdie, 2020; Rafidiyah and Nadia, 2020). Several studies have been conducted for music, drawing, and math students in higher education teaching to overcome the anxiety and stress that is affecting students’ performance in different exams (Ganley et al., 2021; Mendoza et al., 2021; Blair and Van der Sluis, 2022; Caviola et al., 2022). However, others have investigated doctoral students’ learning and life challenges in European countries (Appel and Dahlgren, 2003; Janta et al., 2014; Mattocks and Briscoe-Palmer, 2016; Urrutia et al., 2016). For example, Appel and Dahlgren (2003) conducted a mixed-method study to investigate the financial obstacles encountered by Swedish doctoral students in their doctoral programs. Furthermore, another study also discussed the importance of financial stress on university students’ performance in Bangladesh (Hossain et al., 2022). Hence, prior literature has little discussion on anxiety and stress on doctoral students’ performance through self-regulated learning, which needs to be explored in the present study.

In addition, students encountered challenges such as lack of time for family and leisure activities, and they had doubts about their abilities (e.g., Pyhältö et al., 2012; Mattocks and Briscoe-Palmer, 2016; Hemmati and Mahdie, 2020; Darley, 2021). Mattocks and Briscoe-Palmer (2016) examined the challenges faced by three marginalized groups: women, black minority ethnic groups, and disabled students pursuing a Ph.D. in the United Kingdom; and they encountered challenges in seven different areas: (a) institutional support, (b) finance and funding, (c) confidence and self-esteem, (d) external responsibilities, (e) health and well-being, (f) future professional life, and (g) isolation, exclusion, and disadvantage. Furthermore, several studies added that doctoral students from various disciplines in the UK experienced loneliness and isolation (Janta et al., 2014).

Pyhältö et al. (2012) examined the challenges (e.g., maintaining motivation, self-efficacy beliefs, time management, acquiring expertise in a specific area, problems in supervision, social interaction within the scholarly community, and resources) encountered by 699 doctoral students from different academic backgrounds (e.g., psychology, medicine, philosophy, linguistics, and educational sciences) in their academic research at a Finnish university. Hemmati and Mahdie (2020) examined the experiences of Iranian doctoral students in the learning environment and the challenges they faced in the learning process. Furthermore, Darley (2021) recommended matching doctoral student achievements with resources and competitive advantages, solving the problem of resource shortage, providing guidance and strict supervision regulations, and providing adequate funding for doctoral students.

Moreover, a sort of beneficial, inspiring, and good stress is referred to as eustress (Shafir, 2020). Eustress encourages people to put in extra effort, perform better, and accomplish their goals despite obstacles (Merino et al., 2021). Furthermore, distress refers to the unfavorable stress that most people associate with being “stressed out” (Bak et al., 2022). People in distress frequently feel overburdened and apprehensive and suffer from physical and mental symptoms, including headaches, tension, insomnia, inattentiveness, or impatience (Shafir, 2020). Stress that is frequent, strong, or persistent is harmful to the body and brain, is associated with a range of physical and mental conditions, and impairs one’s capacity to operate. Both eustress and distress include activating the fight or flight response in the body and brain, impacting doctoral students’ performance. The present study covers both aspects and explores whether stress positively or negatively affects doctoral students’ performance.

### 2.3. The impact of self-regulated learning on doctoral students’ performance

Self-regulated learning (SRL) is the process by which individuals convert their mental capacities into academic skills, studying independently and proactively to accomplish the educational objectives they have set for themselves (Barnard et al., 2008, 2009; Panadero, 2017). At the same time, academic performance is a multidimensional construct subdivided into task and contextual performance. Task performance is the capacity to accomplish a job’s primary or central duties (Koopmans et al., 2011). This dimension comprises the capacity for task planning and organization, the focus on results, and the ability for productivity (Widyastuti and Hidayat, 2018). Contextual performance is characterized as additional behavior and activity that goes beyond the primary activities, such as the ability to complete additional tasks, initiative, taking on complicated work, and developing knowledge and skills (Koopmans et al., 2011).

Some researchers have found that effective self-regulating learning strategies benefit doctoral students in learning and completing their assignments (Janta et al., 2014; Kelley and Salisbury-Glennon, 2016; Kumar et al., 2016; Liu and Abliz, 2019). For example, Kelley and Salisbury-Glennon (2016) investigated the impact of self-regulated learning on writing doctoral dissertations. A mixed-methodological, quasi-experimental study with 95 doctoral students found that self-regulating learning strategies helped them make better progress in completing their dissertations and fulfilling their tasks. These students could better meet their tasks and contextual performance with self-regulated learning. Moreover, university students’ academic performance, self-regulated learning techniques, and motivation were all improved by self-regulated learning training programs (Theobald, 2021). Another study investigated the relationship between academic achievement and self-regulated learning and the importance of school engagement in this process (Estévez et al., 2021). Based on the literature, the present study formulated H1 and H2.

*H1*: Self-regulated learning positively and significantly influences doctoral students’ task performance.

*H2*: Self-regulated learning positively and significantly affects doctoral students’ contextual performance.

### 2.4. The impact of anxiety and stress on self-regulated learning

Feelings of tension, trepidation, or fear are characteristics of anxiety and affect university student achievement more broadly (Mendoza et al., 2021). Previous studies also exhibited anxiety as an emotional and physical reaction (Zhang, 2016; Liu and Abliz, 2019; Liu et al., 2019). So, the present study has treated anxiety as a multidimensional construct and measured the doctoral students’ emotional and physical reactions. Furthermore, another independent variable, Stress, results from the interaction between a demanding and challenging situation and the person’s sense of their ability to handle or overcome these difficulties (von Keyserlingk et al., 2022). The present study focused on doctoral students’ study and research-related stress and their abilities to overcome it through self-regulated learning and to enhance their academic performance.

Moreover, effective self-regulating learning techniques have been discovered to help doctoral students manage their anxiety and stress (Janta et al., 2014; Kelley and Salisbury-Glennon, 2016; Liu and Abliz, 2019). Moreover, Johnson et al. (2021) discussed a math intervention created using a self-regulated learning framework, which characterizes self-regulated learners as linked, self-aware, self-determined, strategic, and resilient kids. An intervention that helps students manage their anxiety starts with a problem-solving method and speaking up when necessary to apply helpful strategies is specifically described. For example, Liu and Abliz (2019) investigated 322 doctoral students from different disciplines in China about their sources of anxiety and self-regulation strategies to reduce anxiety. They found that about a third of the participants were physically and mentally anxious and generally feeling under stress. They suggested that dealing with anxiety and self-awareness be crucial before students implement coping strategies; moreover, students reduce anxiety through self-regulation, such as building friendly and supportive relationships with friends and family, communicating and sharing ideas, being proactive, living a regular life, exercising, setting clear goals and working to become more competent; at the same time, students should master the knowledge and skills needed for graduate study to reduce anxiety through self-regulation learning. Therefore, H3 and H4 were developed for this study.

*H3*: Anxiety has a relationship with self-regulated learning.

*H4*: Stress positively and substantially influences self-regulated learning.

### 2.5. The mediating role of self-regulated learning

Over the past couple of decades, many researchers worldwide have examined doctoral students’ anxiety and its impact on their performance (Bolliger and Halupa, 2012; Hwang et al., 2015; Levecque et al., 2017; Liu et al., 2019; Nagy et al., 2019; Jones-White et al., 2020). Another study was conducted to determine if students’ academic help-seeking behavior (self-regulation learning) could be explained by their sense of belonging and academic performance (Won et al., 2021). For example, Levecque et al. (2017) found that many doctoral students experienced mental health problems (i.e., depression and anxiety). The most common manifestations were persistent stress, unhappiness, depression, sleep problems due to anxiety, inability to overcome difficulties, and inability to enjoy daily activities. Hwang et al. (2015) found that many doctoral students had a lot of anxieties, such as research anxiety, statistical anxiety, and library anxiety, which would adversely affect their performance.

More recently, Liu et al. (2019) investigated the mental health status of Chinese medical doctoral students. The results indicated that unbalanced anxiety from family, work, and doctoral programs could cause them much anxiety, resulting in worse mental and even physical health problems. Similarly, Nagy et al. (2019) investigated the mental health problems of biomedical doctoral students in the United States. They found that depression and anxiety could lead to burnout and dropping out of school. Specifically, depression and anxiety could affect their happiness and cause obstacles to their academic performance (Ozer and Akçayoğlu, 2021). Furthermore, effective self-regulating learning techniques were discovered to help doctoral students manage their anxiety and stress (Janta et al., 2014; Kelley and Salisbury-Glennon, 2016). These strategies could also help enhance their academic performance. These findings assisted the present study in formulating H5.

*H5*: Self-regulated learning mediates the relationship between anxiety and doctoral students’ performance.

Several researchers focused on the sources of stress for doctoral students and their impact on their performance (Wilson and Onwuegbuzie, 2001; Zhang, 2016; Barry et al., 2018; Pappa et al., 2020). Wilson and Onwuegbuzie (2001) indicated that the pressure on most doctoral students came from the heavy workload, work difficulty, and formal examination. More specifically, Zhang (2016) conducted focus group interviews with international Chinese doctoral students at American universities regarding their study experience. The results indicated that their stress stemmed from mastering a new language and adapting to a new culture and academic environment. So, stress proved to be a source of the declined work performance of doctoral students (Ganley et al., 2021; Hensley et al., 2021; Hossain et al., 2022).

Furthermore, Janta et al. (2014) found that doctoral students from different disciplines and countries experienced loneliness and a lack of emotional support, which was attributed to a lack of social interaction and an inability to be part of a social group. In addition, they found that doctoral students adopted a series of self-regulating strategies to reduce stress. For example, they (a) interacted with peers by organizing activities with other students, such as lunch, discussing research, or setting up doctoral groups, (b) established doctoral forums, and (c) pursued professional development opportunities, such as teaching opportunities in schools and opportunities to serve as assistant professors, and (d) escaping from academia, e.g., some doctoral students argued that associating with people outside of academia was good for staying sane. These self-regulated learning approaches helped in enhancing the performance of doctoral students. According to the literature, H6 was formulated for this study.

*H6*: Self-regulated learning mediates the relationship between stress and doctoral students’ performance.

To sum up, despite many studies conducted about doctoral students’ learning challenges, there are still gaps in that doctoral students face enormous challenges in task and contextual performance. The implications of anxiety and stress on self-regulated learning are also considered necessary in task and contextual performance. Still, they are not fully assessed, creating a research gap that the current study aimed to fill.

### 2.6. The hypothesized model

**Figure 1:**
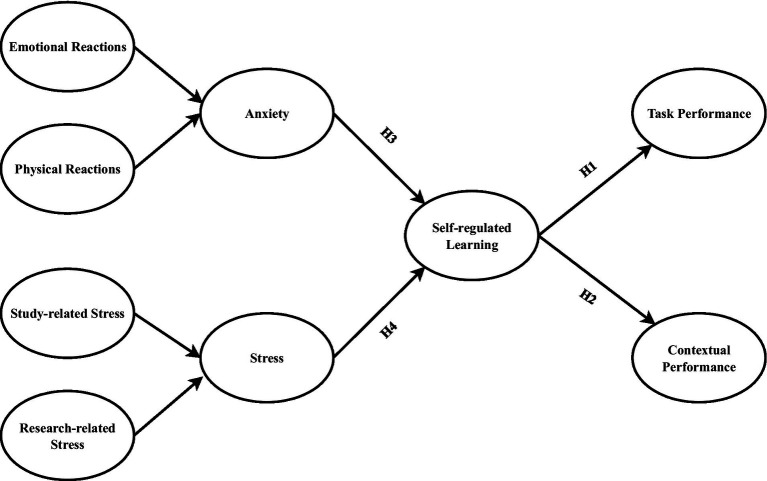
Theoretical framework.

Anxiety and stress are viewed in the proposed model as higher-order latent variables. The resulting model, depicted in Figure 1, assumes that anxiety and stress are independent variables and that self-regulated learning is a mediator. Additionally, this study believes that self-regulated learning plays a crucial role in doctoral students’ performance and, as a result, qualifies as a potent construct. The dependent variable is doctoral students’ performance (task and contextual performance), and self-regulated learning functions as a mediator in the current study.

## 3. Research methods

### 3.1. Participants

A total of 491 doctoral students and recent completers representing 112 universities in China were invited to participate in this study. Recent completers refer to the doctoral students that have graduated no more than 3 years because students who have more than 3 years have less probability of answering the recall-dependent questions. No specific criteria were defined about university selection; and doctoral students from various Chinese universities made up the sample population. The convenience sampling technique was used for gathering data. Among them, 206 (41.9%) were male, and 285 (58.1%) were female participants; 202 (41.1%) majored in arts and 289 (58.9%) in sciences; 237 (48.3%) were currently studying for their doctoral degrees and 254 (51.7%) recently completed their programs and were presently holding a doctoral degree; 171 (34.8%) were aged 30 and younger, and 320 (65.2%) were aged over 30.

### 3.2. The instrument

This study conceptualizes anxiety, the independent variable, as a higher-order formative construct and assesses it using two constructs: emotional reactions and physical reactions (Zung, 1971; Zigmond and Snaith, 1983; Stern, 2014; Liu and Abliz, 2019). Based on research by Liu and Abliz (2019), six items were used to generate emotional reactions. For example, “*I get nervous and anxious easily*.” The Cronbach alpha value for the adapted scale was.93, as shown by the study by Liu and Abliz (2019). Moreover, six items were used to quantify physical reactions from Zigmond and Snaith (1983). For instance, “*My arms and legs shake and tremble*.” Participants were required to indicate their responses on a scale ranging from 1 (strongly disagree) to 5 (strongly agree). The scale was also reliable, validated, and demised by previous studies (Stern, 2014).

Another independent and formative variable, Stress, was also formed and evaluated on two constructs; study-related stress and research-related stress. Six items were used to measure the study-related stress construct obtained from a study by Hong and Huang (2018). For example, “*I feel stressful about not being able to graduate on time*.” Similarly, six items from Hong and Huang (2018) that were modified to measure research-related stress were used. For instance, “*I feel stressful about getting my research article(s) published*.” Participants had to rate their answers on a scale from 1 (strongly disagree) to 5 (strongly agree).

The mediating variable, known as self-regulated learning, was developed by Barnard et al. (2009) and is measured on eight items. For instance, “*I set standards for my learning tasks*.” This variable was a formative construct in the source study, but further, it was demised by other studies and showed reliable findings (Broadbent et al., 2021; Davis and Hadwin, 2021; Won et al., 2021; Yanti et al., 2021). The dependent variable, which measures task performance, is taken from the research of Widyastuti and Hidayat (2018) and is composed of five items. For example, “*I planned my learning tasks so that it was done on time*.” The scale for task performance was reliable, showing a value of 0.871 (Widyastuti and Hidayat, 2018). The other dependent variable, contextual performance, was measured on five items based on the study of Widyastuti and Hidayat (2018). For instance, “*I start new learning tasks when my old ones are completed*.” Participants had to rate their answers on a scale from 1 (strongly disagree) to 5 (strongly agree). The scale of contextual performance was reliable and showed a value of.858 in the source study (Widyastuti and Hidayat, 2018).

The scale was first constructed in English before it was translated into Chinese. The forward and backward translation approach was used for all items to assure the accuracy of the translation (Sperber et al., 1994). Two linguistic professors were invited to translate the questionnaire into Chinese. Then two other professors were invited to translate the Chinese version into English to check the correctness and precision of the scale items. Furthermore, a pilot study was also carried out with the assistance of 25 doctoral students to provide the questionnaire with its final validation. After a successful pilot test, a few minor changes to the questions were made to account for linguistic differences and increase the validity and reliability of the questionnaire. The complete questionnaire with its factor loadings is included in Appendix A.

### 3.3. Data collection procedures

Ethical review approval was obtained before the final data collection. Data collection was conducted online using the Survey Star platform in May 2022. The present study carried out an online survey and shared the link with several WeChat groups of doctoral students in several universities with the help of Ph.D. supervisors. The primary sample of the study was doctoral students, so it was guided and advised that only Ph.D. students could participate in the current research. The researchers provided all the participants with information about the study and consent forms. They all understood that their participation was voluntary and their responses were strictly confidential.

### 3.4. Data analysis methods

Using IBM SPSS 22.0 and Smart PLS 3.0, the following statistical analyses were performed on the survey data: The measurement model analysis, the structural model analysis, the mediation analysis, and the multi-group analysis. The partial least square-structural equation modeling (PLS-SEM) method was employed in this investigation since it is a well-liked method for examining new research trends and developing models rather than only providing confirmation (Urbach and Ahlemann, 2010). PLS-SEM is the best fit over covariance-based-structural equation modeling (CB-SEM) because it simultaneously measures reflecting and formative constructs, which helps solve the constraints mentioned above within the constructs (Gaskin et al., 2018). CB-SEM, however, typically only supports reflecting models. The decision to use PLS-SEM was also based on its capacity to simultaneously estimate causal links among all latent components while addressing measurement errors in the structural model (Hair et al., 2017).

The measurement model analysis was used to check the reliability and validity of the measuring instrument. The measurement model was tested using well-established principles for reliability, average variance extracted (AVE), and discriminant and convergent validity. The reliability of the constructs was initially examined, and the recommended criterion was 0.70 or higher (Hair et al., 2017). The Cronbach alpha (0.70 or higher) shows that the construct is stable and reliable with repeated measurement, as calculated in SmartPLS. The following parameter used in this study was composite reliability (CR). The CRs for all the constructs were investigated, and the cut-off level suggested in the literature was.70 (Hair et al., 2017). The AVE was also used to examine the convergent and divergent validity, and the AVE’s recommended criteria were higher than.50 (Fornell and Larcker, 1981). These values are the standards from literature and different software for reliability, composite reliability, and convergent and divergent validity.

Discriminant validity of the instrument was tested next and defined as that all of the constructs in the instrument should not be substantially correlated (Hubley, 2014), and these conventions were explained by Fornell and Larcker (1981). The heterotrait-Monotrait ratio (HTMT) is another reliable method for evaluating discriminant validity (Henseler et al., 2016) and overcomes the flaws of the Fornell-Larcker criterion, which are less rigorous. To meet the HTMT requirement, all values must be less than 0.90 (Sarstedt et al., 2011; Henseler et al., 2016). The variance inflation factor (VIF) was also determined for each variable. Smart PLS 3.0 has various useful features for measuring the VIF, which assesses the degree of multicollinearity. The VIF values should be less than 3.3 (Petter et al., 2007). It takes a little more finesse to validate higher-order formative constructs. The current research considered the impact of first to second-order factors in the case of these constructs. The goal of this study was to confirm the existence and validation of the two aspects of anxiety and two dimensions of stress (Marakas et al., 2007). The importance of the indicators was first investigated in this study. After the measurement model had been verified as valid and reliable, the structural model was inspected.

Based on the existing literature, different hypotheses were developed to answer the two research questions. Structural model and mediation analyses were applied to answer the first research question (i.e., how will Chinese doctoral students’ self-regulated learning mediate the influence of anxiety and stress on their task performance and contextual performance in their doctoral studies?). Four hypotheses were developed for the structural model analysis. It was hypothesized that self-regulated learning would directly impact task and contextual performance. Moreover, it was further hypothesized that anxiety and stress affect self-regulated learning. A *t*-value higher than 1.196 would indicate that the hypothesis is significant. Additionally, mediation analyses were conducted, explaining the mediation role of self-regulated learning on anxiety and stress-performance relationships. The present study used the most recent conventions to test different constructs’ mediating roles, focusing on bootstrapping (Zhao et al., 2010; Hayes, 2013; Hussain et al., 2021).

Finally, the multi-group analysis (MGA) technique was applied to answer the second research question (i.e., are there significant structural equation modeling differences across demographic variables of gender, major, status, and age?). A significant difference exists between comparing groups in PLS-MGA if the *value of p*s are greater than 0.95 and less than.05 (Sarstedt et al., 2011; Henseler et al., 2016).

## 4. Results

### 4.1. The measurement model analysis

Table 1 explains the constructs’ reliability, CR, and AVE. The reliability of the constructs was found in the recommended criteria. CR for all seven constructs ranged from 0.829 to 0.916, which were higher than the cut-off level suggested in the literature, indicating that the model was convergent. AVE varied from.542 to.663 for all constructs, significantly higher than the cut-off. Moreover, all the items and the standardized factor loadings for each item were included in Appendix A.

**Table 1:** The reliability and construct validity.

Constructs	Cronbach’s alpha	CR	AVE
Emotional reactions	0.889	0.916	0.646
Physical reactions	0.864	0.899	0.600
Study-related stress	0.723	0.829	0.551
Research-related stress	0.897	0.922	0.663
Self-regulated learning	0.879	0.904	0.542
Task performance	0.845	0.891	0.624
Contextual performance	0.858	0.899	0.640

AVE = average variance extracted.

The square root of the average variance retrieved for each construct was higher than the square of the inter-construct correlations. The discriminant validity of the components in the measurement model was further tested, and the results are presented in Table 2.

**Table 2:** The discriminant validity – Fornell-Larcker criterion.

Constructs	Contextual performance	Emotional reactions	Physical reactions	Research-related stress	Self-regulated learning	Study-related stress	Task performance
Contextual performance	*0.80*						
Emotional reactions	−0.084	*0.804*					
Physical reactions	−0.089	0.712	*0.775*				
Research-related stress	0.047	0.397	0.313	*0.814*			
Self-regulated learning	0.734	−0.023	−0.038	0.099	*0.766*		
Study-related stress	−0.014	0.521	0.495	0.668	0.03	*0.68*	
Task performance	0.62	−0.101	−0.057	0.004	0.703	−0.062	*0.79*

Diagonals (italic) values are the square root of the AVE values of each respective construct.

All constructs have an HTMT ratio of less than 0.90; this requirement is also met, as indicated in Table 3. As a result, the constructs’ discriminant validity has been established.

**Table 3:** The HTMT.

	CP	ER	PR	RS	SL	SS	TP
CP							
ER	0.116						
PR	0.131	0.805					
RS	0.13	0.451	0.359				
SL	0.896	0.12	0.136	0.125			
SS	0.166	0.63	0.613	0.777	0.138		
TP	0.724	0.195	0.163	0.109	0.804	0.178	

CP = Contextual performance, ER = Emotional reactions, PR = Physical reactions, RS = Research-related stress, SL = Self-regulated learning, SS = Study-related stress, TP = Task performance.

VIF of all variables was less than the cut-off number to avoid multicollinearity. Furthermore, Table 4 shows the estimated findings with reliable provision for the two dimensions of anxiety and stress as a second-order construct.

**Table 4:** Upward dimension effects of anxiety and stress.

Relationship	Type	Original mean	*T* statistics	*p*-Value
ER → anxiety	1st → 2nd	0.564	46.594	<0.01
PR → anxiety	1st → 2nd	0.516	49.665	<0.01
SS → stress	1st → 2nd	0.669	41.567	<0.01
RS → stress	1st → 2nd	0.421	28.258	<0.01

ER = Emotional reactions, PR = Physical reactions, SS = Study-related stress, RS = Research-related stress.

### 4.2. The structural model analysis

This study explored how anxiety, stress, and self-regulated learning influenced task and contextual performance. Path coefficients were determined after data were obtained to test the hypotheses. Table 5 shows the results of structural equation modeling with PLS for the suggested model. According to the model, adjusted R^2^ was sufficient: R^2^ = 62.9% for CP and R^2^ = 49.3% for TP. At the 99.9% confidence level, all primary routes except anxiety to self-regulated learning were significant. Stress and self-regulated learning had a 62.9% impact on contextual performance, whereas 49.3% on task performance.

**Table 5:** Structural relationships and hypothesis testing.

Hypothesis	Path	Path coefficient	*T* statistics	*p*-values	Decision
H1	SL → TP	0.703	28.540	**	Supported
H2	SL → CP	0.794	41.641	**	Supported
H3	Anxiety→SL	−0.095	1.751	n.s.	Not Supported
H4	Stress→SL	0.126	2.182	**	Supported

**p < 0.01; n.s. = not significant.

**Figure 2:**
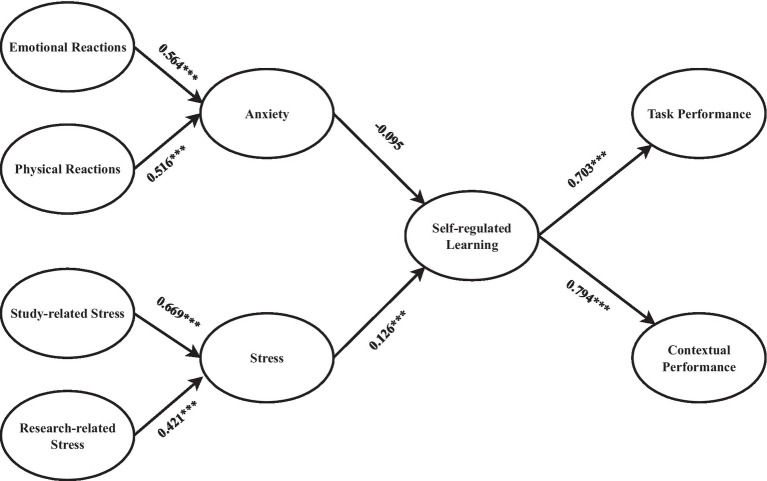
Path coefficients. ***p < 0.01.

About H1, a significant positive relationship existed between SL and TP (β = 0.703, *t* = 28.540, *p* < 0.01). H2 examined the impact of SL on CP. SL significantly influenced the TP (*β* = 0.794, *t* = 41.641, *p* < 0.01). As a result, H2 was endorsed. H3 examined the effects of anxiety on SL. The results showed that there was an insignificant negative relationship between anxiety and SL (*β* = −0.095, *t* = 1.751, *p* > 0.01). About H4, the relationship between stress and SL was significant and favorable (*β* = 0.126, *t* = 2.182, *p* < 0.01). Table 5 and Figure 2 explain all the results for the hypotheses.

### 4.3. The mediation analysis

The present study applied the latest conventions that focus on bootstrapping (Hayes, 2013; Shahbaz et al., 2020). Following these guidelines requires substantial direct and indirect effects (Gaskin et al., 2018). According to the findings, anxiety had an insignificant relationship with TP (*β* = 0.021, *t* = 0.219) and CP (*β* = −0.004, *t* = 0.079). The results showed that anxiety did not have a significant relationship with SL. Therefore, SL was not mediating between anxiety and dependent variables (TP and CP). According to the findings, stress directly affected TP (*β* = −0.083, *t* = 1.976) and CP (*β* = −0.067, *t* = 2.024). The indirect influence of stress on TP and CP *via* SL was also significant. So, SL was a mediator between stress and performance (TP and CP). The present research also calculated variance accounted for (VAF) to determine the magnitude by dividing indirect effect over total effect. Partial mediation exists when the values of VAF lie between 20 and 80% (Hair Jr et al., 2016). Table 6 shows all the mediation results.

**Table 6:** The mediation effects.

Path	Direct effect	Indirect effect	Total effect	*t-*statistics	*p*-Values	VAF	Type of mediation
Stress→TP	0.083			1.976	<0.05		
Stress→SL → TP		0.088	0.171	2.167	<0.05	0.515	Partial
Stress→CP	0.067			2.024	<0.05		
Stress→SL → CP		0.100	0.167	2.153	<0.05	0.599	Partial

TP = Task performance, SL = Self-regulated learning, CP = Contextual performance.

### 4.4. The multi-group analysis

Finally, the current research analyzed whether there were any differences in the effect of anxiety, stress, and SL on TP and CP between gender, major, status, and age studied in this study. As shown in Table 7, the *value of p*s in PLS-MGA were less than.05, indicating the impact of anxiety on SL (*value of p* was 0), SL on CP (*value of p* was 0.005), and stress on SL (*value of p* was.001) differed in Arts and Sciences group. No significant differences were found for the other three groups (i.e., gender, status, and age).

**Table 7:** The multi-group analysis results.

	Path coefficients-diff. major(1.0) – major(2.0)	*Value of p* major(1.0) –major(2.0)
Anxiety →SL	0.452	0
ER → anxiety	0.007	0.779
PR → anxiety	−0.043	0.043
RRS → stress	0.015	0.633
SL → CP	−0.103	0.005
SL → TP	0.028	0.547
SRS → stress	0.007	0.806
Stress →SL	−0.398	0.001

## 5. Discussion and conclusion

### 5.1. Findings of the study

This study’s primary goal and first research question were to formulate and validate the impact of anxiety and stress on doctoral students’ tasks and contextual performance. First, the study confirmed that anxiety was made up of two reflective constructs (i.e., emotional and physical reactions), and stress comprised two reflective constructs (i.e., study-related stress and research-related stress). Anxiety and stress were addressed in the prior studies (Janta et al., 2014; Kelley and Salisbury-Glennon, 2016; Liu and Abliz, 2019), but these constructs were treated as unidimensional constructs. The present study proved that anxiety and stress should be viewed as multidimensional concepts to grasp their full potential for academicians.

Moreover, the PET explains how anxiety broadly impacts performance; specifically, it affects task and contextual performance. In addition, PET also explored that individuals’ (doctoral students in this study) performance are affected by the degree of anxiety (Calvo and Eysenck, 1992) and stress, which is further explored by the present study. The direct impact of anxiety on task and contextual performance was insignificant, whereas the stress had a negative but significant effect on task and contextual performance. It is proved by the present study that anxiety (emotional and physical reactions) would not affect performance. In doctoral studies, the students are assigned different tasks, and due to time constraints, the supervisors do not think about their emotional and physical reactions. They demand that these tasks must be fulfilled in the given period. However, suppose a doctoral student is stressed (study and research-related stress). In that case, it will directly affect his performance, and the present study shows a significant but negative relationship between stress and performance. The prior studies also proved these results (Vincent et al., 2021). Furthermore, eustress and distress are two different concepts applied in the context of the present study. Stress has a two-way aspect; it can be beneficial and inspiring, referred to as eustress, but the current research results differentiate it from the concept of eustress. Moreover, the other form of stress is distress, and the present study complies with this concept because Chinese doctoral students felt stressed out, which impacted their performance.

Second, the findings revealed that self-regulated learning considerably affected task and contextual performance. This finding was consistent with previous research, which indicated that self-regulated learning significantly impacted students’ performance (Kumar et al., 2016). As a result, doctoral students with self-regulated solid learning strategies can enhance their task and contextual performance. Third, the model demonstrated that stress positively impacted self-regulated learning. Students were primarily facing study-related and research-related stress in their doctoral studies. So, this stress created a positive relationship with self-regulated learning, as proved in the prior literature (e.g., Liu and Abliz, 2019). Doctoral students attempted to cope with the stress by regulating their self-learning, which eventually could improve their performance.

Fourth, the present study discovered that anxiety had an insignificant impact on self-regulated learning. As a multidimensional construct, anxiety was formulated on emotional and physical reactions. The current study proved that emotional and physical reactions did not impact self-regulated learning. Doctoral students who are not in a position to control their emotions and physical reactions cannot be self-regulated learners.

Another role of the PET was also to testify to the importance of self-regulatory mechanisms (Calvo and Eysenck, 1992). So, the present study followed the PET and checked the mediating role of self-regulated learning, leading to the second research question. The model and findings indicated that self-regulated learning partially mediated stress and doctoral students’ performance (task and contextual performance). It showed that doctoral students’ study and research-related stress negatively impacted their task and contextual performance. However, if they cope with self-regulated learning strategies, they can manage the study and research-related stress and improve their performance, as shown in the current study.

Finally, the current research further analyzed the third research question about any differences between gender, major, status, and age. The results showed significant differences in major (arts and sciences group) between three central relationships (anxiety on SL, SL on CP, and stress on SL). The sample included doctoral students doing arts majors, 41 and 59% were science majors. Both majors are entirely different in terms of theoretical and experimental work; and the present study has provided similar results with literature (Vitasari et al., 2010; Pyhältö et al., 2012). So, the multi-group analysis showed that both arts and science majors had significant differences. No significant differences were found for the other three groups (i.e., gender, status, and age).

### 5.2. Limitations of the study

This study was limited in the following three ways that must be acknowledged. First, this study focused on the mediating impact of self-regulated learning on doctoral students’ performance; it did not evaluate the moderating effect. Second, this study did not incorporate cultural values into the research design. It is believed that culture can significantly influence how students perceive themselves. Thirdly, the convenience sample approach was used in this study due to time and resource limitations. However, future studies can overcome this restriction by utilizing any alternative sampling strategy. Forth, limited sociodemographic factors were considered to measure the differences between the opinions about major, gender, status, and age. Future research on household income, marital status, and urban vs. rural area students can be conducted. Finally, this study adopted a quantitative research approach. The use of qualitative methods could help validate the quantitative findings. All these limitations could limit the interpretation and generalization of the results.

### 5.3. Conclusion

In light of these limitations, the following four conclusions are drawn. First, based on the literature, the present study formulated three formative constructs, i.e., anxiety, stress, and academic performance, and the results proved those as formatives. Second, based on self-regulated learning, the present study framed and investigated a mediation model to capture the influence of anxiety and stress on doctoral students’ tasks and contextual performance. This empirical investigation showed that self-regulated learning was a necessary and binding force between stress and doctoral students’ performance. Third, it showed that stress was the most crucial factor and positively influenced and enhanced self-regulated learning, improving students’ tasks and contextual performance. Finally, this study emphasized the relevance and significance of students’ self-regulated learning and identified it as a cutting-edge concept for boosting their performance in their doctoral studies.

### 5.4. Implications

The results of this study would have implications for researchers, academicians, doctoral students’ supervisors, industry, and government that want to reduce anxiety and stress among doctoral students and assist them in improving their performance in doctoral studies. Research on anxiety and stress will help researchers to understand the problem better and equip them to handle it because doctoral students differ from one another in various aspects such as gender, age, program, goal, research training and ability, relationship with friends, family members, and supervisors, among others. Anxiety is a multidimensional construct in this study, comprised of emotional and physical reactions. So, humor, training, and other interventions can help doctoral students lessen anxiety. Furthermore, stress reduction strategies like psychoeducation and relaxation training should be implemented.

Last but not least, the current study showed that self-regulated learning was crucial for enhancing doctoral students’ performance. Therefore, it is the responsibility of doctoral students to develop their attitudes and habits toward self-regulated learning. Also, policymakers and curriculum developers should uniquely design the curriculum to develop self-regulated learning in graduate and doctoral students.

## Data availability statement

The original contributions presented in the study are included in the article/supplementary material, further inquiries can be directed to the corresponding author.

## Ethics statement

The studies involving human participants were reviewed and approved by the Evidence-based Research Center for Educational Assessment (ERCEA) Research Ethical Review Board, Jiangsu University. The patients/participants provided their written informed consent to participate in this study.

## Author contributions

ZL: conceptualization, literature, data acquisition, writing, and funding acquisition. JH: conceptualization, literature, methodology, data acquisition, data analysis support, writing—subsequent drafts, reviewing, revising, editing, proofreading, and final draft, preparation and editing for submission. SH: conceptualization, literature, methodology, data analysis, writing, reviewing, revising, editing, and proofreading. TS: literature, data acquisition, writing, and revising. All authors contributed to the article and approved the submitted version.

## Funding

This study was funded by the Introduction of Talent Research Start-up Fund at Zhejiang Fashion Institute of Technology in 2021. Project Number: 13001104004011.

## Conflict of interest

The authors declare that the research was conducted in the absence of any commercial or financial relationships that could be construed as a potential conflict of interest.

## Publisher’s note

All claims expressed in this article are solely those of the authors and do not necessarily represent those of their affiliated organizations, or those of the publisher, the editors and the reviewers. Any product that may be evaluated in this article, or claim that may be made by its manufacturer, is not guaranteed or endorsed by the publisher.

## Appendix A

Measure items and factor loadings.

**Table:**

Items	Description	Factor loadings
ER1	I get nervous and anxious easily.	0.730
ER2	I get upset easily.	0.826
ER3	I feel like something awful is about to happen.	0.788
ER4	I have nightmares.	0.736
ER5	I get sudden feelings of panic.	0.866
ER6	I feel afraid for no reason at all.	0.867
PR1	My arms and legs shake and tremble.	0.704
PR2	I am bothered by headaches and neck and back pains.	0.663
PR3	I feel weak and get tired easily.	0.798
PR4	I can feel my heart beating fast.	0.869
PR5	I am bothered by dizzy spells.	0.833
PR6	I am bothered by stomach and indigestion.	0.760
SS1	I feel stressful about not being able to graduate on time.	0.777
SS2	I feel stressed about meeting my doctoral supervisor’s expectations.	0.831
SS3	I feel stressed about participating in research activities.	0.741
SS4	I feel stressed about participating in social activities.	0.610
SS5	I feel stressed about participating in family activities.	0.600
SS6	I feel stressed about finding employment.	0.514
RS1	I feel stressful about getting my research article(s) published.	0.740
RS2	I feel stressed about determining my doctoral dissertation topic.	0.769
RS3	I feel stressed about designing my doctoral dissertation study.	0.893
RS4	I feel stressed about successfully defending my dissertation proposal.	0.759
RS5	I feel stressed about writing my dissertation.	0.862
RS6	I feel stressed about successfully defending my dissertation.	0.852
SL1	I set standards for my learning tasks.	0.770
SL2	I set goals for my learning tasks.	0.813
SL3	I have a daily schedule for my study.	0.708
SL4	I finish my learning tasks according to my schedules	0.729
SL5	I seek help from my peers when I need it.	0.641
SL6	I seek help from my professors when I need it.	0.718
SL7	I self-evaluate my learning regularly.	0.745
SL8	I communicate regularly with my supervisor about my progress.	0.754
TP1	I planned my learning tasks so that it was done on time.	0.804
TP2	I set priorities to complete my learning tasks.	0.849
TP3	I kept in mind the outcomes that I had to achieve in my learning.	0.806
TP4	I was able to separate main issues from side issues in my learning.	0.858
TP5	I could complete my learning tasks well with minimal time and effort.	0.607
CP1	I start new learning tasks when my old ones are completed.	0.784
CP2	I take on challenging learning tasks when they are available.	0.837
CP3	I work hard to keep my research skills up-to-date.	0.849
CP4	I continually seek new challenges in my study.	0.808
CP5	I actively participate in research meetings and discussions.	0.715

ER = Emotional reactions; PR = Physical reactions; SS = Study-related stress; RS = Research-related stress; SL = Self-regulated learning; TP = Task performance; CP = Contextual performance.
